# Effects of granulocyte-colony stimulating factor (G-CSF) on diabetic cardiomyopathy in Otsuka Long-Evans Tokushima Fatty rats

**DOI:** 10.1186/1475-2840-10-92

**Published:** 2011-10-17

**Authors:** Young-Hyo Lim, Jun-Ho Joe, Ki-Seok Jang, Yi-Sun Song, Byung-Im So, Cheng-Hu Fang, Jinho Shin, Jung-Hyun Kim, Heon-Kil Lim, Kyung-Soo Kim

**Affiliations:** 1 Division of Cardiology, Department of Internal Medicine, College of Medicine, Hanyang University, Heangdang-Dong 17, Seungdong-ku, Seoul, Korea; 2 Department of Biomedical Sciences, College of Medicine, Hanyang University, Heangdang-Dong 17, Seungdong-ku, Seoul, Korea; 3 Department of Pathology, College of Medicine, Hanyang University, Heangdang-Dong 17, Seungdong-ku, Seoul, Korea

**Keywords:** Diabetes Mellitus, Cardiomyopathy, Echocardiography, Doppler, Histology, Fibrosis

## Abstract

**Background:**

Diabetic cardiomyopathy (CMP) is a common and disabling disease in diabetic patients, however no effective treatments have been developed. Although granulocyte-colony stimulating factor (G-CSF) improves heart function in myocardial infarction, its effect on non-ischemic CMP such as diabetic CMP is unknown. In the present study, we investigated the effects of G-CSF on diabetic CMP in a rat model of type II diabetes.

**Methods:**

Twenty 7-week-old male Otsuka Long-Evans Tokushima Fatty (OLETF: a rat model of diabetes) rats and 10 male Long-Evans Tokushima Otsuka (LETO: normal controls) rats were used. All of the LETO and 8 OLETF rats were fed on tap water while the rest were fed on sucrose-containing water. After 10 weeks, saline or recombinant human G-CSF (100 μg/kg/day) was injected intraperitoneally for 5 days. Blood levels of glucose, total cholesterol and triglyceride, and Doppler echocardiograms for diastolic dysfunction were obtained just before and 4 weeks after the saline or G-CSF treatment. Light microscopy, electron microscopy (EM) and immunohistochemistry for transforming growth factor-β were employed to examine myocardial histology 4 weeks after the saline or G-CSF treatment.

**Results:**

Diastolic dysfunction developed at 17 weeks (before the saline or G-CSF treatment) in the OLETF rats whether or not they were fed sucrose water, but were more severe in those fed sucrose water. Four weeks after saline or G-CSF treatment, diastolic function had recovered in the G-CSF-treated group regardless of sucrose water feeding, and perivascular and/or interstitial fibrosis in the G-CSF-treated group had decreased significantly. TGF-β immunoreactivity in the interstitial and perivascular tissue was also reduced in the G-CSF-treated group, and EM studies revealed less severe disruption of myofilaments and mitochondrial cristae, and decreased collagen deposition.

**Conclusions:**

G-CSF can ameliorate cardiac diastolic dysfunction and morphological damage, especially fibrosis of the myocardium, in OLETF rats with diabetic CMP.

## Introduction

Diabetic cardiomyopathy (CMP) is defined as cardiac failure when possible etiologies such as alcohol, hypertension, and coronary and structural heart disease have been excluded in diabetic patients [1]. In a previous study using type II diabetes mellitus (DM) rats model, diabetic CMP was characterized functionally by the presence of left ventricular diastolic dysfunction, and histologically by interstitial fibrosis and collagen accumulation [2]. Diabetic CMP has been reported in 30-75% of diabetic patients, and is an important cause of heart failure [3-7]. The worldwide prevalence of diabetes has been estimated to increase from 2.8% in 2000 to 4.4% in 2030, this could lead to an increase of diabetic CMP [8]. Although coronary artery diseases are the main cause of heart failure and deteriorating function, the high incidence of diabetic CMP indicates that diabetes itself is an important factor in myocardial damage. There are currently no clearly established treatments for diabetic CMP [9,10]. Conventional therapeutic practice, such as strict control of blood glucose level and avoidance of traditional risk factors, is often effective, but does not completely prevent cardiac complications [11-13]. Therefore, there is a great need to develop effective treatments for diabetic CMP.

Recent studies have shown that granulocyte-colony stimulating factor (G-CSF) reduces the size of infarcts, induces myocardial regeneration and is responsible for recovery of heart function in myocardial infarction (MI) [14-17]. Based on these reports, we hypothesized that G-CSF might also have beneficial effects on non-ischemic CMP, such as diabetic CMP. Therefore, in the present study, we investigated the functional and histological effects of G-CSF on diabetic cardiomyopathy in Otsuka Long-Evans Tokushima Fatty (OLETF) rats.

## Methods

### Subjects

This work was performed in compliance with the ARRIVE guidelines on animal research [18]. We used as experimental subjects twenty male Otsuka Long-Evans Tokushima Fatty (OLETF) rats; these rats were developed as spontaneous long-term hyperglycemic rats with type II DM [19]. Ten male Long-Evans Tokushima Otsuka (LETO) rats, which were developed from the same colony by selective mating but do not develop DM, were used as control animals. All the rats were kept in the specific pathogen-free facility of the Hanyang University Medical School Animal Experiment Center at controlled temperature (23 ± 2°C) and humidity (55 ± 5%) with a 12-hour artificial light and dark cycle. All rats care and experimental procedures were performed according to the guidelines of Hanyang University for Experiments in Animals, and all protocols were approved by the Hanyang University Institutional Review Board.

### Experimental protocols

**Figure 1:**
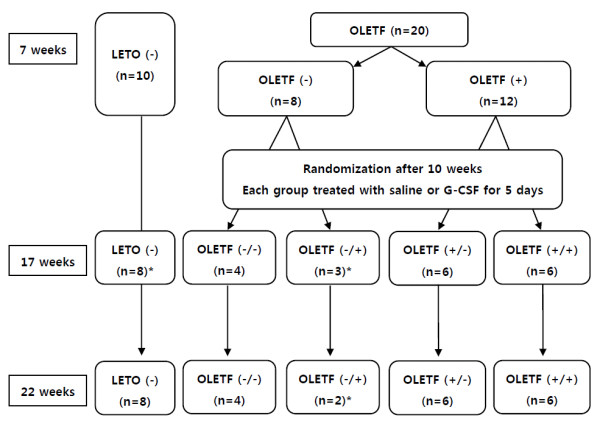
**Brief experimental protocol**. LETO; Long-Evans Tokushima Otsuka rat, OLETF; Otsuka Long-Evans Tokushima Fatty rat, G-CSF; granulocyte-colony stimulating factor, BW; body weight, BP; blood pressure, HR; Heart rate. LETO (-); LETO rats fed on tap water, OLETF (-); OLETF rats fed on tap water, OLETF (+); OLETF rats fed on sucrose contained water, OLETF (-/-); OLETF rats fed on tap water and treated with saline, OLETF (-/+); OLETF rats fed on tap water and treated with G-CSF, OLETF (+/-); OLETF rats fed on sucrose water and treated with saline, OLETF (+/+); OLETF rats fed on sucrose water and treated with G-CSF. * Rats expired during blood pressure measurement

The numbers of rats in each group of LETO and OLETF rats and a schema of the experimental protocol are given in Figure 1. This experiment started at rat age of 7 weeks. The rats were given free access to standard laboratory rat chow, all of the LETO (LETO (-)) and 8 randomly selected OLETF rats were given free access to tap water (OLETF (-)), and other 12 OLETF rats were given free access to water containing 30% sucrose (OLETF (+)). At rat age of 17 weeks (after 10 weeks), each OLETF rat groups was divided randomly into two groups, which were injected, respectively, with saline or recombinant human G-CSF (100 μg/kg/day; Dong-A Pharmacological, Seoul, Korea) intraperitoneally for 5 days. Thus there were eventually 5 groups of rats: LETO rats fed on tap water (LETO (-)), OLETF rats fed on tap water and treated with saline (OLETF (-/-)), OLETF rats fed on tap water and treated with G-CSF (OLETF (-/+)), OLETF rats fed on sucrose water and treated with saline (OLETF (+/-)) and OLETF rats fed on sucrose water and treated with G-CSF (OLETF (+/+)).

Body weight, fasting blood glucose (FBG), total cholesterol (TC) and triglyceride (TG), heart rate (HR), systolic blood pressure (SBP) were measured, and Doppler echocardiography performed, at two times: first at the age of 17 weeks (just before treated) and second at the age of 22 weeks (4 weeks after the 5-day intraperitoneal saline or G-CSF injection). After the rats were killed, myocardial histology was examined with light microscopy, immunohistochemistry for transforming growth factor (TGF)-β and electron microscopy (EM).

SBP was measured by the tail-cuff method (Sphygmomanometer PS-100 Rick, Riken Kaihatsu). Blood tests were measured in tail capillary blood after 8 hours of fasting.

### Echocardiography

Each rat was anesthetized with 50 mg/kg ketamine and 5.8 mg/kg xylazine HCl, and the left side of the chest was shaved to gain a clear image. Doppler echocardiography was performed by a single sonographer. Serial echocardiographic examinations (Philips iE33, Philips Medical System with an S8-3 probe) were performed with the rats in the left lateral decubitus position. The transmitral flow velocity profile was determined by positioning a sample volume at the tip of the mitral valve on the apical four chamber view. The Doppler beam was set with < 15° of the incident angle to flow direction identified on color Doppler image. Angle correction was performed by a semiautomated system installed in the machine. The peak velocity (E), deceleration time (DT) of the early diastolic filling wave and early mitral annulus velocity (E') were measured. DT was obtained by extrapolating the initial slope of early diastolic filling wave deceleration to the baseline. E' was measured at the septal portion of the mitral annulus in an apical four chamber view, using tissue Doppler technique with a Nyquist limit of 15 cm/s. The transmitral inflow pattern was recorded on a strip chart at 100 mm/s sweep speed with simultaneous 3-lead electrocardiography for offline analysis. All measurements represented the mean of 5 consecutive cardiac cycles, and heart rate was calculated on the basis of the strip chart of Doppler echocardiography.

### Euthanasia and Histopathological Methods

Rats were anesthetized with 50 mg/kg ketamine and 5.8 mg/kg xylazine HCl. The hearts were excised and separated into 2 halves along the anterior longitudinal middle line. One half of the heart was fixed with formalin solution, embedded in paraffin and cut into sections 4 μm thick for H&E, Masson's trichrome staining and immunostaining for the TGF-β receptor (SC-146, Santa Cruz Biotechnology, dilution 1:100). The other half of each heart was frozen in liquid nitrogen and stored at 280°C for electron microscopic examination. Interstitial fibrosis and perivascular fibrosis were measured, using Image Pro plus 6.0 imaging software (Media Cybernetics, Silver Spring, MD) and the results were expressed as percentages.

### Electron microscopy

For electron microscopic examination, a portion of the left ventricle was cut into 1 mm fragments and fixed in 2.5% glutaraldehyde (0.2 M cacodylate buffer, pH 7.4) for 4 hours and in 1% buffered sodium tetroxide for 1 hour, and then embedded in Epon 812. Survey sections were cut at 1 μm and stained with toluidine blue. Ultrathin sections were cut in from this block and studied under a Hitachi H-7600S electron microscope at 80 KV.

### Statistical Analysis

All values are means ± standard deviation (SD). Statistical differences were determined with Statistical Package for the Social Sciences (SPSS) 18.0 software (SPSS Inc., Chicago, IL, USA). Data were analyzed by *t*-tests (for single comparisons) or one-way ANOVA (for multiple comparisons), and Post Hoc multiple comparisons were made with Tukey's test (equal variances assumed) or Tamhane's T2 test (equal variances not assumed). p values of less than 0.05 were considered significant.

## Results

### 1) Establishment of diabetic cardiomyopathy

Table 1 presents baseline data on the three groups of rats at 17 weeks, before treatment with G-CSF. Body weight and blood level of TG were significantly higher in the OLETF groups than the LETO (-). However, blood levels of FBG, and TC did not differ between the OLETF and LETO (-) groups. Also no significant differences in blood levels of FBG, TC and TG were observed between the two OLETF groups.

**Table 1:** Baseline data before treatment with G-CSF or saline (at the time of 17 weeks old)

	LETO (-) (n = 8)	OLETF (-) (n = 7)	OLETF (+) (n = 12)	p
**BW (g)**	**428 ± 18.8**	551.4 ± 44.9*	595.7 ± 37.5*‡	**< 0.001**
**FBG (mg/dl)**	**125.4 ± 9.8**	**148.0 ± 28.5**	**151.3 ± 34.5**	**0.129**
**TC (mg/dl)**	**71.9 ± 19.8**	97.3 ± 25.0**	**89.8 ± 15.1**	**0.045**
**TG (mg/dl)**	**46.6 ± 23.4**	197.7 ± 91.2**	190.7 ± 41.6*	**< 0.001**
**HR (bpm)**	**312.9 ± 18.7**	**297.6 ± 24.5**	**298.8 ± 12.2**	**0.177**
**E (cm/sec)**	**72.8 ± 3.3**	66.3 ± 1.7**	65.5 ± 5.4*	**0.002**
**E' (cm/sec)**	**9.0 ± 1.4**	6.7 ± 0.2*	5.2 ± 0.3*†	**< 0.001**
**E/E'**	**8.2 ± 1.0**	9.8 ± 0.4*	12.5 ± 0.8*†	**< 0.001**
**DT (msec)**	**81.8 ± 12.3**	**83.6 ± 9.5**	**86.3 ± 11.4**	**0.675**

Values are mean ± SD. *p < 0.01 and **p < 0.05 versus LETO (-). †p < 0.01 and ‡p < 0.05 versus OLETF (-).

LETO; Long-Evans Tokushima Otsuka rat, OLETF; Otsuka Long-Evans Tokushima Fatty rat, BW; body weight, FBG; fasting blood glucose TC; total cholesterol, TG; triglyceride, HR; Heart rate, E; peak velocity of the early diastolic filling wave, E'; early mitral annulus velocity, DT; deceleration time, LETO (-); LETO rats fed on tap water, OLETF (-); OLETF rats fed on tap water, OLETF (+); OLETF rats fed on sucrose contained water

E and E' were lower and E/E' was higher in the two OLETF groups than in the LETO (-) rats, showing that diastolic dysfunction had developed in the OLETF rats. Moreover the OLETF (+) rats had lower E' and higher E/E' than the OLETF (-) rats; thus diastolic dysfunction was more pronounced in the sucrose-fed OLETF rats. In that, diabetic CMP models using OLETF rats were comparatively well made when compared with age-matched LETO rats group, using diastolic function parameters by Doppler echocardiography.

### 2) G-CSF improves cardiac diastolic function

Doppler echocardiography was performed four weeks after the series of intraperitoneal injections of G-CSF or normal saline. In the tap water groups, E' showed a higher trend (p = 0.062) and E/E' was lower (p < 0.05) in the G-CSF treated (OLETF (-/+)) rats than the saline-treated (OLETF (-/-)) rats. This shows that G-CSF had a protective effect on diastolic function. Furthermore, there were no significant differences of E, E' and E/E' between OLETF (-/+) and LETO (-), indicating that G-CSF increased diastolic function to a level similar to that in the control rats. (Table 2)

**Table 2:** Data of LETO rats and OLETF rats fed on tap water at the time of 22 weeks old (4 weeks after G-CSF or saline treatment)

	LETO (-) (n = 8)	OLETF (-/-) (n = 4)	OLETF (-/+) (n = 2)	p
**BW (g)**	**444.0 ± 15.2**	600.2 ± 31.2*	537.5 ± 95.5**	**< 0.001**
**FBG (mg/dl)**	**210.8 ± 46.6**	364.0 ± 120.0**	**281.0 ± 89.1**	**0.025**
**TC (mg/dl)**	**55.4 ± 6.9**	**61.5 ± 7.6**	**61.0 ± 12.7**	**0.397**
**TG (mg/dl)**	**45.9 ± 12.5**	151.0 ± 62.0*	162.0 ± 46.7*	**0.001**
**HR (bpm)**	**292.9 ± 18.1**	**283.5 ± 28.3**	**291.0 ± 24.0**	**0.785**
**E (cm/sec)**	**79.5 ± 4.2**	**73.6 ± 2.2**	**75.2 ± 0.4**	**0.051**
**E' (cm/sec)**	**7.1 ± 0.5**	5.7 ± 0.3*	**6.7 ± 0.4**	**0.001**
**E/E'**	**11.2 ± 0.6**	12.9 ± 0.7*	**11.3 ± 0.7‡**	**0.004**
**DT (msec)**	**76.4 ± 5.5**	**68.5 ± 12.8**	**73.5 ± 0.7**	**0.311**
**BP (mmHg)**	**160.0 ± 14.1**	**167.2 ± 10.1**	**167.4 ± 34.8**	**0.950**

Values are mean ± SD. *p < 0.01 and **p < 0.05 versus LETO (-). †p < 0.01 and ‡p < 0.05 versus OLETF (-/-).

LETO; Long-Evans Tokushima Otsuka rat, OLETF; Otsuka Long-Evans Tokushima Fatty rat, G-CSF; granulocyte-colony stimulating factor, BW; body weight, FBG; fasting blood glucose, TC; total cholesterol, TG; triglyceride, HR; Heart rate, E; peak velocity of the early diastolic filling wave, E'; early mitral annulus velocity, DT; deceleration time BP; blood pressure,

LETO (-); LETO rats fed on tap water,

OLETF (-/-); OLETF rats fed on tap water and treated with saline,

OLETF (-/+); OLETF rats fed on tap water and treated with G-CSF

A more clear-cut set of findings was obtained in the two groups of OLETF rats fed on sucrose-containing water. The differences between the OLETF rats injected with G-CSF (OLETF (+/+)) and those injected with normal saline (OLETF (+/-)) were more prominent, with E' higher and E/E' lower in the OLETF (+/+) compared with the OLETF (+/-) rats. Moreover, E' and E/E' did not differ between the OLETF (+/+) and LETO (-) rats, indicating that G-CSF treatment also improved diastolic function to a level comparable with that in normal rats. (Table 3)

**Table 3:** Data of LETO rats and OLETF rats fed on sucrose contained water at the time of 22 weeks old (4 weeks after G-CSF or saline treatment)

	LETO (-) (n = 8)	OLETF (+/-) (n = 6)	OLETF (+/+) (n = 6)	p
**BW (g)**	**444.0 ± 15.2**	624.0 ± 23.8*	598.7 ± 50.8*	**< 0.001**
**FBG (mg/dl)**	**210.8 ± 46.6**	367.3 ± 47.9*	314.0 ± 56.2*	**< 0.001**
**TC (mg/dl)**	**55.4 ± 6.9**	**58.5 ± 9.7**	**54.2 ± 16.5**	**0.790**
**TG (mg/dl)**	**45.9 ± 12.5**	116.3 ± 16.4*	128.2 ± 55.9**	**< 0.001**
**HR (bpm)**	**292.9 ± 18.1**	**291.7 ± 35.5**	**289.5 ± 18.6**	**0.968**
**E (cm/sec)**	**79.5 ± 4.2**	70.1 ± 1.4*	73.9 ± 3.3**	**0.001**
**E' (cm/sec)**	**7.1 ± 0.5**	4.7 ± 0.1*	**6.6 ± 0.2†**	**< 0.001**
**E/E'**	**11.2 ± 0.6**	15.0 ± 0.7*	**11.3 ± 0.8†**	**< 0.001**
**DT (msec)**	**76.4 ± 5.5**	**69.3 ± 7.7**	**72.0 ± 4.0**	**0.105**
**BP (mmHg)**	**160.0 ± 14.1**	**166.9 ± 2.0**	**177.0 ± 21.9**	**0.587**

Values are mean ± SD. *p < 0.01 and **p < 0.05 versus LETO (-). †p < 0.01 and ‡p < 0.05 versus OLETF (+/-).

LETO; Long-Evans Tokushima Otsuka rat, OLETF; Otsuka Long-Evans Tokushima Fatty rat, G-CSF; granulocyte-colony stimulating factor, BW; body weight, FBG; fasting blood glucose, TC; total cholesterol, TG; triglyceride, HR; heart rate, E; peak velocity of the early diastolic filling wave, E'; early mitral annulus velocity, DT; deceleration time, BP; blood pressure,

LETO (-); LETO rats fed on tap water,

OLETF (+/-); OLETF rats fed on sucrose water and treated with saline,

OLETF (+/+); OLETF rats fed on sucrose water and treated with G-CSF

**Figure 2:**
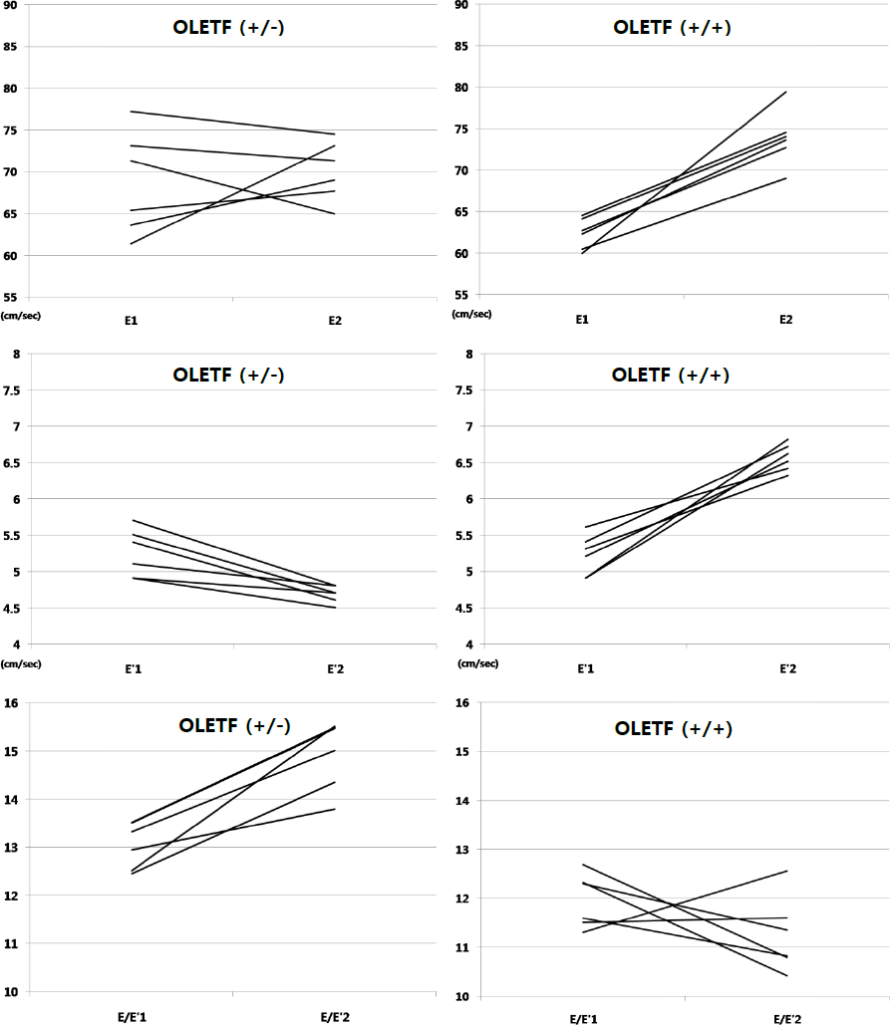
**Changes in diastolic parameters of individual subjects of the OLETF rats fed on sucrose contained water group; OLETF rats treated with saline (OLETF (+/-)) (left) and OLETF rats treated with G-CSF (OLETF (+/+) (right)**. E; peak velocity of the early diastolic filling wave, E'; early mitral annulus velocity, E1, E'1, E/E'1:pre-treatments state, E2, E'2, E/E'2: four weeks after treatments.

The changes in E, E' and E/E' of OLETF rats fed on sucrose-containing water and then injected with saline of G-CSF are shown in Table 4 and Figure 2. E' increased significantly and E/E' was maintained in the OLETF (+/+), while E' decreased and E/E' increased significantly in the OLETF (+/-). In those treated with saline (OLETF (+/-)), most of the subjects showed a decline in E' and a rise in E/E'. In contrast, in those treated with G-CSF (OLETF (+/+)), an increase in E' and a decrease in E/E' was seen in most of the subjects. These results mean that diastolic function was preserved or even improved in the rats treated with G-CSF.

**Table 4:** Changes of echocardiographic data pre- and post-treatment of OLETF rats fed on sucrose contained water

	OLETF (+/-)			OLETF (+/+)		
	
	Pre- treatment	Post- treatment	p	Pre- treatment	Post- treatment	p
**E (cm/sec)**	**68.7 ± 6.1**	**70.1 ± 1.4**	**0.611**	**62.4 ± 1.8**	**73.9 ± 3.3**	**0.001**
**E' (cm/sec)**	**5.3 ± 0.3**	**4.7 ± 0.1**	**0.006**	**5.2 ± 0.3**	**6.6 ± 0.2**	**0.001**
**E/E'**	**13.0 ± 0.5**	**15.0 ± 0.7**	**0.001**	**12.0 ± 0.6**	**11.3 ± 0.8**	**0.224**

Values are mean ± SD.

OLETF; Otsuka Long-Evans Tokushima Fatty rat, G-CSF; granulocyte-colony stimulating factor, E; peak velocity of the early diastolic filling wave, E'; early mitral annulus velocity

OLETF (+/-); OLETF rats fed on sucrose water and treated with saline,

OLETF (+/+); OLETF rats fed on sucrose water and treated with G-CSF

### 3) G-CSF prevents histological damage to the myocardium in the diabetic rats

**Figure 3:**
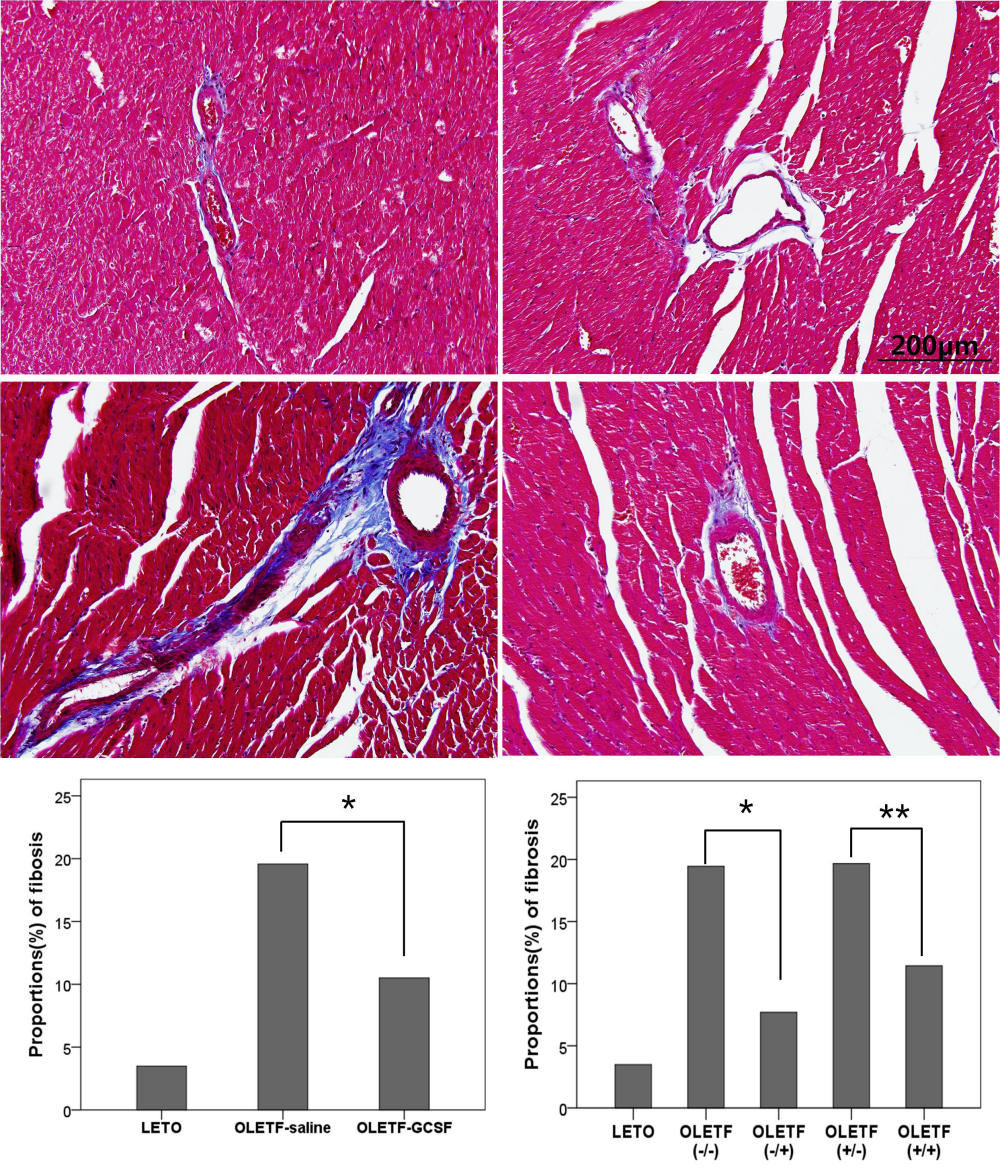
**The representative sections for Masson trichrome staining OLETF groups**.(*200). The perivascular and interstitial fibrosis is demonstrated as blue color on Masson Trichrome stain. Proportion of fibrosis (bottom) is significantly decreased in G-CSF treated OLETF rat (E, F: Image Pro analysis). * p < 0.05, ** p < 0.01. A. OLETF (-/-); OLETF rat fed on tap water and treated with saline, B. OLETF (-/+); OLETF rat fed on tap water and treated with G-CSF, C. OLETF (+/-); OLETF rat fed on sucrose water and treated with saline, D. OLETF (+/+); OLETF rat fed on sucrose water and treated with G-CSF.

Masson trichrome staining revealed less perivascular and interstitial fibrosis in the OLETF rats treated with G-CSF than in those treated with saline. In the Image-pro analysis, the extent of fibrosis was greater in the OLETF groups than in the LETO group, and significantly lower in the G-CSF- treated group than the saline-treated group. In subgroup analysis, both groups of OLETF rats treated with G-CSF displayed less fibrosis than those treated with saline. (Figure 3)

**Figure 4:**
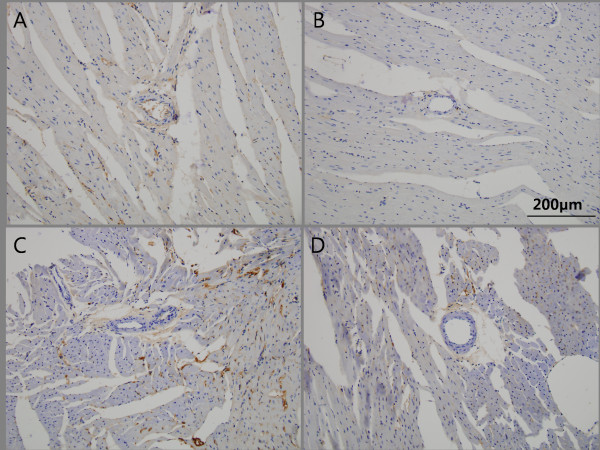
**The representative sections of immunohistochemical staining for TGF-β in non-treated and G-CSF treated OLETF rats**. Tissues expressing TGF-β appeared brown color. (*200). The minimal interstitial staining for TGF-β is found in OLETF (-/-), whereas complete absence in OLETF (-/+). Similarly, the extent of TGF-β immunoreactivity in the interstitial and perivascular tissue is significantly decreased in OLETF (+/+) compared with OLETF (+/-).A. OLETF (-/-); OLETF rat fed on tap water and treated with saline, B. OLETF (-/+); OLETF rat fed on tap water and treated with G-CSF, C. OLETF (+/-); OLETF rat fed on sucrose water and treated with saline, D. OLETF (+/+); OLETF rat fed on sucrose water and treated with G-CSF.

Similarly only a low level of interstitial immunostaining for TFG-β was observed in the OLETF (-/-) rats, and a complete absence of staining in the OLETF (-/+) rats. Likewise, the level of TGF-β immunoreactivity in interstitial and perivascular tissue was significantly lower in the OLETF (+/+) rats than in the OLETF (+/-) rats. (Figure 4)

**Figure 5:**
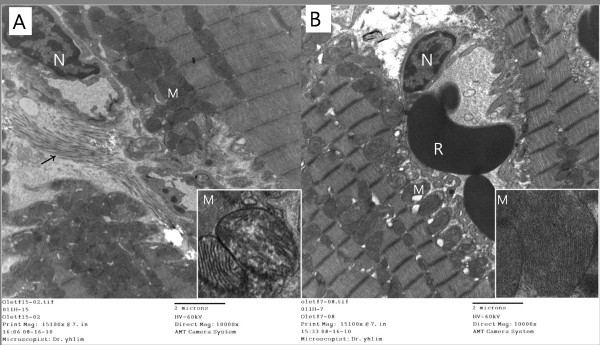
**The ultrastructure of saline-treated sucrose contained water fed OLETF rat (OLETF (+/-)) myocardium displays deposition of collagen fibers in pericapillary region and swelling of mitochondria in cardiac cells, whereas G-CSF treat rats (OLETF (+/+)) reveals absence of collagen deposition and relatively decreased number of abnormal mitochondria**. Note the disruption or distortion of cristae network of the enlarged mitochondria in non-treated sucrose fed group (inset). N: nucleus, R: red blood cell, M: mitochondria, arrow: collagen fiber. A. OLETF (+/-); OLETF rat fed on sucrose water and treated with saline, B. OLETF (+/+); OLETF rat fed on sucrose water and treated with G-CSF.

Electron microscopy revealed more pronounced heterogeneous structural alterations in the myocardial tissue of the OLETF (+/-) rats than in that of the OLETF (+/+) rats. Ultrastructurally the OLETF (+/-) myocardium showed the presence of intracellular edema, deposition of collagen fibers in the pericapillary region, swelling and disrupted mitochondrial cristae, and damaged myofilaments and intracellular junctions, as well as increased accumulation of lipid droplets and lysosomes in the cardiac cells, In contrast, collagen deposition was absent from the OLETF (+/+) myocardium and there were fewer abnormal mitochondria. Moreover there was less evidence of disruption of the cristae of enlarged mitochondria, or of damaged myofilaments and intracellular junctions. (Figure 5)

## Discussion

In the diabetic heart, the functional alterations are preceded by a variety of structural, molecular and cellular changes, many of which are present in asymptomatic diabetic individuals and experimental models of diabetes. Moreover, the undermining of myocardial and vascular integrity appears to begin during the pre-diabetic stage [20]. In a recent study, changes in cardiac heparin sulfate proteoglycan expression and the diastolic dysfunction, an early sign of diabetic CMP occurred as parallel events. Although several pathophysiological mechanisms have been proposed to explain the diabetic CMP, it remains poorly understood [20,21].

Another study reported that in an MI model, hyperglycemia had a beneficial effect on delaying ischemic-reperfusion injury and left ventricular dysfunction, however hyperglycemia did not decrease mortality rate [22], and diabetic CMP is a very important cause of heart failure [3-7].

In this study we evaluated the functional and histological effect of G-CSF on relatively early diabetic CMP. We observed no substantial differences in blood levels of FBG, TC and TG between G-CSF-treated and saline-treated OLETF rats, suggesting that G-CSF does not significantly affect glucose and lipid metabolism.

In diabetic CMP, diastolic dysfunction is well on the way even before symptoms appear in the patient. Therefore, we evaluated E, E', DT and E/E' using Doppler echocardiography and tissue Doppler to assess the degree of diastolic dysfunction. We found that diastolic dysfunction was minimal or completely recovered in OLETF rats treated with G-CSF.

The beneficial effects of G-CSF were shown in the histologic findings as well. We found that perivascular and interstitial fibrosis progressed more slowly or even showed improvement in G-CSF treated OLETF rat groups. Also, the extent of TGF-β immunoreactivity in the interstitial and perivascular tissue was significantly decreased in G-CSF treated OLETF rat groups. EM studies revealed decreased collagen deposition and lesser number of abnormal mitochondria, damaged myofilaments and intracellular junctions were also noted in G-CSF treated OLETF rats. Our results indicate that the protective effects of G-CSF are seen not only in relatively advanced diabetic CMP but also in its early stages.

Although various animal models have been used to investigate diabetes-related changes in the myocardium including interstitial fibrosis, cardiomyocyte loss, impaired energy utilization, small vessel disease and neuropathy, the pathophysiology of diabetic cardiomyopathy remains to be fully elucidated [10]. The present study provides the first evidence of the beneficial effects of G-CSF on diabetic CMP in OLETF rats, an animal model of non-ischemic CMP. The two major findings of this study were that G-CSF delayed a progression of diastolic dysfunction, in terms of function, and prevented interstitial and perivascular fibrosis, in terms of pathology. Until now the beneficial effect of G-CSF on the heart has been mainly studied in the myocardial infarction model. Although there have been a few studies of the effect of G-CSF on non-ischemic CMP, studies on the effect of G-CSF on diabetic CMP are limited [23,24].

G-CSF has various functions such as induction of proliferation, survival and differentiation of hematopoietic cells and mobilization of bone marrow cells [25-27]. It has been reported that bone marrow cells can differentiate into cardiomyocytes and vascular cells, thereby contributing to the regeneration of myocardium and to angiogenesis in ischemic hearts [28,29]. According to recent reports, G-CSF reduces the size of infarcts, induces myocardial regeneration and contributes to the recovery of heart function. To account for the effects of G-CSF it has been proposed that it promotes the mobilization of bone marrow stem cells or acts directly on cardiomyocytes of the injured heart, however the precise mechanisms of the beneficial effects of G-CSF are not fully understood [14-17]. The therapeutic effects of G-CSF have been also described in non-ischemic dilated cardiomyopathies such as idiopathic dilated CMP or doxorubicin-induced CMP [23,24].

Harada et al. have discussed the possible molecular mechanisms of G-CSF action in preventing left ventricular remodeling after MI. They found that G-CSF acted directly on a G-CSF receptor expressed on cardiomyocytes and activated the Jak/Stat pathway. G-CSF also induced antiapoptotic proteins and inhibited apoptotic death of cardiomyocytes [14]. Baldo MP et al. recently reported that G-CSF preserved left ventricular function through early infarct size reduction and the attenuation of cardiac remodeling after MI via anti-apoptotic protein upregulation and an early reduction in cardiomyocyte apoptosis [15]. Minatoguchi et al. hypothesized that G-CSF accelerate the healing of MI wounds and they reported that it accelerated absorption of necrotic tissues by increasing macrophage numbers, and reduced granulation and scar tissue by promoting the expression of matrix metalloproteinases [16]. Hamamoto et al. reported that G-CSF promoted the migration of bone marrow cells to diseased hearts and also directly enhanced the proliferation of troponin I-positive cardiomyocytes via G-CSF receptors on the cardiomyocytes [23]. Although many proposals regarding the mechanism of the protective effects of G-CSF on myocardium have been reported, the exact mechanism is yet to be established [14-17].

In the present study, we found in the immunohistochemical findings that G-CSF inhibited presentation of TGF-β and progression of fibrosis. Such findings can enhance our understanding of the effects of G-CSF on myocardium, though further investigation is required to establish the exact mechanism of the protective effect of G-CSF in diabetic CMP.

### • Limitation of the study

This study has two main limitations. First the number of subjects was small. Second, data on optimal G-CSF regimens such as dose, timing, frequency and duration are limited. In order to overcome these limitations, a preclinical study of the effects of G-CSF on diabetic CMP should be carried out with a larger number of subjects, and the G-CSF regimen should be optimized. Elucidating the major mechanism underlying the improvement of heart function by G-CSF treatment will require much more investigation.

### • Clinical and research implications of the findings

In the clinical setting, patients with diabetic CMP have increased in number, and moreover end-stage dilated CMP is the reality. Despite the increasing burden of diabetic CMP, the development of new treatment modalities has received little attention, and treatment is still largely based on conventional approaches such as strict glycemic control, management of symptoms and the use of renin-angiotensin-aldosterone system inhibitors and beta blockers [9-12]. In this study, we demonstrated that G-CSF has beneficial functional and histological effects on diabetic CMP. Further studies on the mechanism and for clinical implications would be desirable.

## Conclusions

In this study we evaluated the functional and histological effect of G-CSF on relatively early diabetic CMP. We found that diastolic dysfunction was minimal or completely recovered in OLETF rats treated with G-CSF. The beneficial effects of G-CSF were shown in the histologic findings as well. Perivascular and interstitial fibrosis progressed more slowly or even showed improvement in G-CSF treated OLETF rat groups. Also, the extent of TGF-β immunoreactivity in the interstitial and perivascular tissue was significantly decreased in G-CSF treated OLETF rat groups. EM studies revealed decreased collagen deposition and lesser number of abnormal mitochondria, damaged myofilaments and intracellular junctions were also noted in G-CSF treated OLETF rats. Our results indicate that the protective effects of G-CSF are seen diabetic. Therefore, G-CSF can ameliorate cardiac diastolic dysfunction and morphological damage, especially fibrosis of the myocardium, in OLETF rats with diabetic CMP. The present study provides the first evidence of the beneficial effects of G-CSF on diabetic CMP in OLETF rats.

## List of Abbreviations

CMP: cardiomyopathy; DM: diabetes mellitus; G-CSF: granulocyte-colony stimulating factor; OLETF: Otsuka Long-Evans Tokushima Fatty; LETO: Long-Evans Tokushima Otsuka; FBG: fasting blood glucose; TC: total cholesterol; TG: triglyceride; HR: heart rate; SBP: systolic blood pressure; TGF: transforming growth factor; EM: electron microscopy; E: peak velocity of the early diastolic filling wave; DT: deceleration time; E': early mitral annulus velocity; SD: standard deviation; SPSS: Statistical Package for the Social Sciences

## Competing interests

The authors declare that they have no competing interests.

## Authors' contributions

YHL designed, coordinated the experiments, performed the echocardiographic measurements, and prepared the manuscript. JHJ participated in the immunohistochemistry and helped with the echocardiography measurements. KSJ coordinated and studied the pathology findings. YSS and CHF helped with the echocardiography measurements. BIS participated in the electron microscopy. JS helped to draft the manuscript. JHK and HKL participated in the design and data discussion. KSK conceived the study and participated in the design and coordination. All authors have read and approved the final manuscript.

## References

[B1] Rubler S Dlugash J Yuceoglu YZ Kumral T Branwood AW Grishman A New type of cardiomyopathy associated with diabetic glomerulosclerosis Am J Cardiol 1972 30 6 595 602 10.1016/0002-9149(72)90595-4 4263660

[B2] Mizushige K Yao L Noma T Kiyomoto H Yu Y Hosomi N Ohmori K Matsuo H Alteration in left ventricular diastolic filling and accumulation of myocardial collagen at insulin-resistant prediabetic stage of a type II diabetic rat model Circulation 2000 101 8 899 907 10694530 10.1161/01.cir.101.8.899

[B3] Takenaka K Sakamoto T Amano K Oku J Fujinami K Murakami T Toda I Kawakubo K Sugimoto T Left ventricular filling determined by Doppler echocardiography in diabetes mellitus Am J Cardiol 1988 61 13 1140 1143 10.1016/0002-9149(88)90149-X 3364373

[B4] Robillon JF Sadoul JL Jullien D Morand P Freychet P Abnormalities suggestive of cardiomyopathy in patients with type 2 diabetes of relatively short duration Diabete Metab 1994 20 5 473 480 7859895

[B5] Boyer JK Thanigaraj S Schechtman KB Perez JE Prevalence of ventricular diastolic dysfunction in asymptomatic, normotensive patients with diabetes mellitus Am J Cardiol 2004 93 7 870 875 10.1016/j.amjcard.2003.12.026 15050491

[B6] Zabalgoitia M Ismaeil MF Anderson L Maklady FA Prevalence of diastolic dysfunction in normotensive, asymptomatic patients with well-controlled type 2 diabetes mellitus Am J Cardiol 2001 87 3 320 323 10.1016/S0002-9149(00)01366-7 11165968

[B7] Wachter R Luers C Kleta S Griebel K Herrmann-Lingen C Binder L Janicke N Wetzel D Kochen MM Pieske B Impact of diabetes on left ventricular diastolic function in patients with arterial hypertension Eur J Heart Fail 2007 9 5 469 476 10.1016/j.ejheart.2007.01.001 17303471

[B8] Wild S Roglic G Green A Sicree R King H Global prevalence of diabetes: estimates for the year 2000 and projections for 2030 Diabetes Care 2004 27 5 1047 1053 10.2337/diacare.27.5.1047 15111519

[B9] Marwick TH Diabetic heart disease Heart 2006 92 3 296 300 16159978 10.1136/hrt.2005.067231 PMC1860832

[B10] Fang ZY Prins JB Marwick TH Diabetic cardiomyopathy: evidence, mechanisms, and therapeutic implications Endocr Rev 2004 25 4 543 567 10.1210/er.2003-0012 15294881

[B11] Eckel J Reinauer H Insulin action on glucose transport in isolated cardiac myocytes: signalling pathways and diabetes-induced alterations Biochem Soc Trans 1990 18 6 1125 1127 1965168 10.1042/bst0181125

[B12] Gotzsche O Myocardial cell dysfunction in diabetes mellitus A review of clinical and experimental studies Diabetes 1986 35 10 1158 1162 10.2337/diabetes.35.10.1158 3530845

[B13] Garvey WT Hardin D Juhaszova M Dominguez JH Effects of diabetes on myocardial glucose transport system in rats: implications for diabetic cardiomyopathy Am J Physiol 1993 264 3 Pt 2 H837 844 8456985 10.1152/ajpheart.1993.264.3.H837

[B14] Harada M Qin Y Takano H Minamino T Zou Y Toko H Ohtsuka M Matsuura K Sano M Nishi J Iwanaga K Akazawa H Kunieda T Zhu W Hasegawa H Kunisada K Nagai T Nakaya H Yamauchi-Takihara K Komuro I G-CSF prevents cardiac remodeling after myocardial infarction by activating the Jak-Stat pathway in cardiomyocytes Nat Med 2005 11 3 305 311 10.1038/nm1199 15723072

[B15] Baldo MP Davel AP Damas-Souza DM Nicoletti-Carvalho JE Bordin S Carvalho HF Rodrigues SL Rossoni LV Mill JG The Antiapoptotic Effect of Granulocyte Colony-stimulating Factor Reduces Infarct Size and Prevents Heart Failure Development in Rats Cell Physiol Biochem 2011 28 1 33 40 10.1159/000331711 21865846

[B16] Minatoguchi S Takemura G Chen XH Wang N Uno Y Koda M Arai M Misao Y Lu C Suzuki K Goto K Komada A Takahashi T Kosai K Fujiwara T Fujiwara H Acceleration of the healing process and myocardial regeneration may be important as a mechanism of improvement of cardiac function and remodeling by postinfarction granulocyte colony-stimulating factor treatment Circulation 2004 109 21 2572 2580 10.1161/01.CIR.0000129770.93985.3E 15123535

[B17] Adachi Y Imagawa J Suzuki Y Yogo K Fukazawa M Kuromaru O Saito Y G-CSF treatment increases side population cell infiltration after myocardial infarction in mice J Mol Cell Cardiol 2004 36 5 707 710 10.1016/j.yjmcc.2004.03.005 15135666

[B18] Kilkenny C Browne WJ Cuthill IC Emerson M Altman DG Improving Bioscience Research Reporting: The ARRIVE Guidelines for Reporting Animal Research PLoS Biol 2010 8 6 e1000412 10.1371/journal.pbio.1000412 20613859 PMC2893951

[B19] Kawano K Hirashima T Mori S Saitoh Y Kurosumi M Natori T Spontaneous long-term hyperglycemic rat with diabetic complications. Otsuka Long-Evans Tokushima Fatty (OLETF) strain Diabetes 1992 41 11 1422 1428 10.2337/diabetes.41.11.1422 1397718

[B20] Asghar O Al-Sunni A Khavandi K Khavandi A Withers S Greenstein A Heagerty AM Malik RA Diabetic cardiomyopathy Clin Sci (Lond) 2009 116 10 741 760 10.1042/CS20080500 PMC2782307 19364331

[B21] Strunz CM Matsuda M Salemi VM Nogueira A Mansur AP Cestari IN Marquezini MV Changes in cardiac heparan sulfate proteoglycan expression and streptozotocin-induced diastolic dysfunction in rats Cardiovasc Diabetol 2011 10 35 10.1186/1475-2840-10-35 21518435 PMC3100243

[B22] Rodrigues B Rosa KT Medeiros A Schaan BD Brum PC De Angelis K Irigoyen MC Hyperglycemia can delay left ventricular dysfunction but not autonomic damage after myocardial infarction in rodents Cardiovasc Diabetol 2011 10 26 10.1186/1475-2840-10-26 21470409 PMC3084163

[B23] Hamamoto M Tomita S Nakatani T Yutani C Yamashiro S Sueda T Yagihara T Kitamura S Granulocyte-colony stimulating factor directly enhances proliferation of human troponin I-positive cells derived from idiopathic dilated cardiomyopathy through specific receptors J Heart Lung Transplant 2004 23 12 1430 1437 10.1016/j.healun.2003.09.031 15607674

[B24] Tomita S Ishida M Nakatani T Fukuhara S Hisashi Y Ohtsu Y Suga M Yutani C Yagihara T Yamada K Kitamura S Bone marrow is a source of regenerated cardiomyocytes in doxorubicin-induced cardiomyopathy and granulocyte colony-stimulating factor enhances migration of bone marrow cells and attenuates cardiotoxicity of doxorubicin under electron microscopy J Heart Lung Transplant 2004 23 5 577 584 10.1016/j.healun.2003.06.001 15135374

[B25] Avalos BR Molecular analysis of the granulocyte colony-stimulating factor receptor Blood 1996 88 3 761 777 8704229

[B26] Demetri GD Griffin JD Granulocyte colony-stimulating factor and its receptor Blood 1991 78 11 2791 2808 1720034

[B27] Berliner N Hsing A Graubert T Sigurdsson F Zain M Bruno E Hoffman R Granulocyte colony-stimulating factor induction of normal human bone marrow progenitors results in neutrophil-specific gene expression Blood 1995 85 3 799 803 7530510

[B28] Orlic D Kajstura J Chimenti S Jakoniuk I Anderson SM Li B Pickel J McKay R Nadal-Ginard B Bodine DM Leri A Anversa P Bone marrow cells regenerate infarcted myocardium Nature 2001 410 6829 701 705 10.1038/35070587 11287958

[B29] Kocher AA Schuster MD Szabolcs MJ Takuma S Burkhoff D Wang J Homma S Edwards NM Itescu S Neovascularization of ischemic myocardium by human bone-marrow-derived angioblasts prevents cardiomyocyte apoptosis, reduces remodeling and improves cardiac function Nat Med 2001 7 4 430 436 10.1038/86498 11283669

